# Integrating Patient-Reported Outcomes into Atrial Fibrillation Care Pathways: Implementation Challenges, Health System Implications, and Future Directions

**DOI:** 10.3390/healthcare14131904

**Published:** 2026-06-30

**Authors:** Emma Sokolova, Sevinc Elif Sen, Olav Goetz, Daiga Behmane, Oskars Kalējs

**Affiliations:** 1 Department of Doctoral Studies, Riga Stradiņš University, LV-1007 Riga, Latvia; 2 Centre of Clinical Diagnostics, Riga East Clinical University Hospital, LV-1038 Riga, Latvia; 3 Independent Researcher, D02 PN40 Dublin, Ireland; sevincelifalkan@gmail.com; 4 Faculty of Health Economics, APOLLON University of Applied Sciences, 28195 Bremen, Germany; 5 Faculty of Public Health and Social Welfare, Riga Stradiņš University, LV-1007 Riga, Latvia; 6 Latvian Centre of Cardiology, Pauls Stradiņš Clinical University Hospital, LV-1002 Riga, Latvia

**Keywords:** integrated care, patient-reported outcome measures, PROMs, healthcare systems, implementation science, quality of life, value-based healthcare, digital health, AF-CARE pathway, health policy

## Abstract

**Background/Objectives**: Atrial fibrillation (AF) imposes a substantial long-term clinical and healthcare system burden, recurrent hospitalizations, impaired quality of life, and increasing long-term healthcare costs. Although patient-reported outcome measures (PROMs) are increasingly used in AF research and clinical practice, their broader role in healthcare delivery, implementation, and system-level decision-making remains insufficiently defined. Existing assessment strategies frequently prioritize symptom burden while underrepresenting cognitive, emotional, social, and functional dimensions of AF-related impairment. This narrative implementation review examines the current role of PROMs in AF management from a healthcare system and implementation perspective. **Methods**: Literature addressing AF-specific and generic PROM instruments, implementation strategies, health system integration, value-based care, and digital health approaches was reviewed and synthesized across PubMed, Scopus, and Google Scholar. Particular emphasis was placed on implementation barriers, workflow integration, evidence strength, and challenges encountered across diverse healthcare settings. **Results**: Current PROM frameworks incompletely capture several important dimensions of AF burden, including cognitive dysfunction, sleep disturbance, emotional distress, social participation, sexual health, and productivity loss. Beyond conventional symptom assessment, PROMs may support longitudinal patient monitoring, treatment evaluation, shared decision-making, and patient-centred care. Emerging evidence also suggests potential roles in outpatient prioritization, healthcare quality assessment, and value-based healthcare initiatives, although prospective AF-specific implementation studies remain limited. Mapping PROM applications to the 2024 ESC AF-CARE pathway demonstrates the strongest alignment with the Evaluation and Reducing symptoms domains while supporting patient engagement, comorbidity management, and individualized care planning. Implementation remains constrained by clinician workload, questionnaire fatigue, limited interoperability, heterogeneous digital infrastructure, and variability in organizational resources, with these challenges potentially being more pronounced in smaller or resource-limited healthcare systems. **Conclusions**: PROM integration in AF care may provide opportunities to strengthen patient-centered management and improve healthcare system responsiveness beyond conventional rhythm- and symptom-focused approaches. Successful implementation may require careful adaptation to local healthcare infrastructure, workflow feasibility, and long-term sustainability. Future developments involving digital platforms, wearable technologies, and artificial intelligence-assisted interpretation may further expand the clinical and operational relevance of PROM-guided AF care.

## 1. Introduction

Cardiac arrhythmias represent one of the most common manifestations of cardiovascular disease and remain an important cause of morbidity, healthcare utilization, impaired functional status, and reduced health-related quality of life (HRQoL). Among these disorders, atrial fibrillation (AF) is the most prevalent sustained cardiac arrhythmia worldwide, affecting more than 60 million individuals and placing a substantial clinical and economic burden on healthcare systems [1,2,3,4]. The prevalence of AF continues to increase owing to population ageing, improved survival from cardiovascular diseases, and the growing prevalence of cardiovascular risk factors and multimorbidity [2,3,4,5]. Consequently, AF has become a major public health challenge associated with an increased risk of stroke, heart failure, cognitive impairment, recurrent hospitalization, and premature mortality [2,3,4,5,6].

Over the past decade, substantial advances have been achieved in the diagnosis and management of AF through earlier rhythm-control strategies, catheter ablation, optimized anticoagulation, and integrated multidisciplinary care [2,6,7,8,9]. These developments have significantly improved conventional clinical outcomes, including rhythm control, stroke prevention, and survival. Nevertheless, many patients continue to experience persistent symptoms, impaired physical functioning, psychological distress, treatment burden, and reduced quality of life despite apparently successful clinical management [9,10,11,12]. Importantly, the relationship between objective measures of disease severity and patient-reported symptom burden is often weak. Patients with comparable rhythm status, AF burden, or echocardiographic findings may report markedly different experiences regarding daily functioning, emotional well-being, and overall treatment benefit [10,11,12,13].

These observations have contributed to a progressive shift toward patient-centred cardiovascular care, where treatment success is evaluated not only by traditional clinical endpoints but also by outcomes that are meaningful to patients themselves [2,11,14]. Within this framework, patient-reported outcome measures (PROMs) have emerged as standardized and validated instruments for assessing symptoms, functional capacity, health-related quality of life, treatment satisfaction, and the broader impact of disease from the patient’s perspective [11,12,13,14,15]. Rather than replacing conventional clinical assessment, PROMs complement physician-reported outcomes by capturing dimensions of health that are frequently overlooked by electrocardiography, imaging, laboratory biomarkers, or clinical risk scores alone [11,13,14,15,16].

The clinical relevance of PROMs is particularly evident in atrial fibrillation. Symptom severity frequently correlates poorly with objective rhythm characteristics, while psychological well-being, sleep quality, fatigue, cognitive function, and social participation may substantially influence patients’ perception of treatment success [10,11,12,13,14,15,16,17]. Consequently, contemporary AF management increasingly recognizes that effective care should extend beyond rhythm control and stroke prevention to include systematic assessment of patient-reported health status throughout the disease course. This evolving perspective is reflected in the 2024 European Society of Cardiology Guidelines for the Management of Atrial Fibrillation, which incorporate patient-centred care within the AF-CARE framework and recognize PROMs as valuable tools for comprehensive patient evaluation, shared decision-making, longitudinal follow-up, and assessment of treatment effectiveness [2].

Despite growing acceptance of PROMs within contemporary cardiovascular care, important challenges remain regarding their implementation in routine clinical practice. Although several AF-specific instruments—including the Atrial Fibrillation Effect on Quality-of-Life (AFEQT) questionnaire and the Atrial Fibrillation Severity Scale (AFSS)—have demonstrated good psychometric performance and clinical utility [13,14,15,16,17], their integration into everyday healthcare systems remains inconsistent. Considerable heterogeneity exists regarding instrument selection, timing of assessment, digital integration, interpretation of PROM data, and incorporation into clinical workflows. Furthermore, many proposed healthcare system applications—including longitudinal monitoring, healthcare quality assessment, outpatient prioritization, and value-based healthcare—continue to rely predominantly on observational evidence, implementation studies, and expert consensus rather than prospective implementation trials [18,19,20,21,22,23,24,25].

Existing reviews have primarily focused on the psychometric validation of PROM instruments or their association with clinical outcomes. Comparatively less attention has been devoted to the practical challenges of implementing PROMs within contemporary AF care pathways, including organizational barriers, digital health integration, multidisciplinary care models, healthcare system implications, and alignment with the recently introduced AF-CARE framework. As healthcare systems increasingly adopt patient-centred and value-based approaches to chronic disease management, a comprehensive synthesis of these implementation-related aspects has become both timely and clinically relevant.

Although several narrative and systematic reviews have examined patient-reported outcome measures in cardiovascular disease and atrial fibrillation, these publications have primarily focused on instrument validation, psychometric performance, or associations between PROMs and clinical outcomes. Comparatively little attention has been devoted to the broader implementation challenges associated with integrating PROMs into routine AF care or to their potential role within contemporary healthcare systems.

The present review was designed to address this gap by adopting an implementation-oriented perspective. Specifically, this review differs from previous publications in four important respects. First, it examines PROM implementation through the lens of implementation science, emphasizing organizational barriers, facilitators, and real-world clinical integration rather than questionnaire validation alone. Second, it considers the broader healthcare system implications of PROM use, including outpatient prioritization, healthcare quality assessment, digital health, and value-based healthcare. Third, it expands the traditional symptom-oriented perspective by examining hidden dimensions of AF burden, including emotional well-being, cognitive function, fatigue, sleep, and social participation. Finally, this review interprets the current evidence within the framework of the 2024 ESC AF-CARE model, providing a contemporary perspective on how PROMs may contribute to patient-centred atrial fibrillation management while explicitly distinguishing evidence-supported applications from future implementation opportunities.

Accordingly, rather than providing another review of PROM instruments, the present manuscript offers a narrative implementation review that critically evaluates how patient-reported outcomes can be translated from measurement tools into clinically meaningful components of integrated AF care.

Therefore, the aim of this narrative implementation review is to critically evaluate the current evidence regarding the integration of patient-reported outcome measures into atrial fibrillation care pathways, with particular emphasis on implementation challenges, health system implications, digital health, value-based healthcare, and future research priorities within the context of the 2024 ESC AF-CARE framework.

## 2. Materials and Methods

### 2.1. Study Design

This study was designed as a narrative implementation review examining the role of patient-reported outcome measures (PROMs) in atrial fibrillation (AF) care from a healthcare systems and real-world implementation perspective. The review focused not only on the clinical utility of PROMs, but also on their potential operational relevance for longitudinal disease management, outpatient workflow organization, healthcare quality evaluation, and value-based care models. Throughout this review, the term PROM implementation refers to the systematic incorporation of patient-reported outcome measures into routine atrial fibrillation care, whereas PROM-guided care denotes the use of PROM data to inform individual clinical decision-making.

Given the heterogeneity of existing literature in this field, a narrative review methodology was considered more appropriate than a formal systematic review. The available evidence includes a combination of clinical studies, quality-of-life investigations, implementation analyses, healthcare policy papers, digital health reports, and conceptual frameworks, many of which differ substantially in methodology and outcome definitions. The present review therefore aimed to provide an integrative and clinically oriented synthesis rather than a quantitative pooled analysis.

A narrative implementation review design was selected because the objective of the manuscript was not to estimate a pooled effect size, but to integrate evidence across clinically and methodologically diverse domains, including PROM validation studies, quality-of-life research, implementation science, healthcare policy, digital health, and value-based care. A systematic review or meta-analysis was not considered appropriate because the included literature varied substantially in study design, population, outcome definitions, implementation context, and level of empirical evidence.

### 2.2. Literature Search Strategy

**Figure 1:**
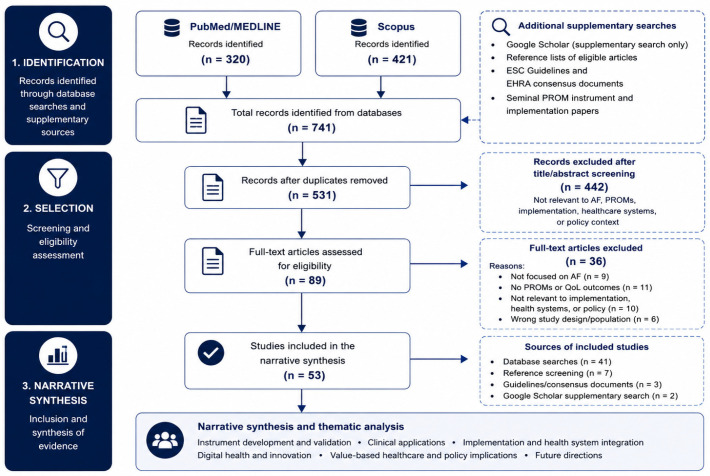
Narrative literature search and study selection process. The review used an iterative narrative search strategy based on structured searches of PubMed/MEDLINE and Scopus, complemented by supplementary searches in Google Scholar, manual reference screening, guideline documents, and seminal publications. The selection process was designed to identify clinically relevant empirical, methodological, implementation, and policy literature rather than to perform a formal systematic review. Arrows indicate the sequential progression of the literature identification, screening, eligibility assessment, and narrative synthesis process, while the boxes summarize the corresponding stages and study counts.

A structured literature search was conducted in PubMed/MEDLINE and Scopus as the primary bibliographic databases. Google Scholar was used exclusively as a supplementary search source to identify recently published articles, grey literature where appropriate, and additional relevant publications through citation tracking. Database-specific search strategies combined terms related to atrial fibrillation, patient-reported outcome measures (PROMs), quality of life, healthcare implementation, patient-centered care, digital health, remote monitoring, value-based healthcare, and health policy. Searches were limited to publications in English published between 1 January 2000 and 31 March 2025. The overall literature identification and narrative study selection process is summarized in Figure 1, while the complete database-specific search strategies are provided in Supplementary Table S1. The primary database search identified 320 records in PubMed/MEDLINE and 421 records in Scopus using the predefined search strategy. Duplicate records retrieved across databases were removed before title and abstract screening. Because this study was designed as a narrative implementation review rather than a systematic review, the primary database search was complemented by targeted supplementary searches. Google Scholar was not used as a primary bibliographic database for study identification; instead, it served as a supplementary source to identify recently published articles, grey literature where appropriate, and additional relevant publications through citation tracking. Reference lists of eligible articles, ESC Guidelines, EHRA consensus documents, and seminal publications describing AF-specific PROM instruments were also manually screened to identify additional studies considered relevant to the objectives of this review. The search process was iterative. Initial searches focused on validated PROM instruments and quality-of-life assessment in atrial fibrillation, whereas subsequent targeted searches identified literature addressing implementation science, healthcare systems, digital health, remote monitoring, artificial intelligence, and value-based healthcare. This approach was considered appropriate because the objective of the review was to synthesize evidence across clinically and methodologically heterogeneous domains rather than to perform a quantitative evidence synthesis.

Rather than relying on a single highly sensitive search, the literature search was performed iteratively using complementary search streams addressing distinct thematic domains relevant to the objectives of this narrative implementation review. Duplicate records identified across databases were removed manually before screening. Titles and abstracts were screened for relevance by the lead author, and potentially eligible full-text articles were assessed in detail. Eligibility decisions were verified by a second author. Disagreements regarding inclusion were resolved through discussion until consensus was reached. Approximately 280 records were identified across database and manual searches; after duplicate removal and screening for relevance, 53 sources were included in the final narrative synthesis. The final selection was based on relevance to the predefined thematic objectives of the review, methodological diversity, contribution to implementation science, and clinical applicability to contemporary AF care pathways, while avoiding duplication of evidence across multiple publications.

The primary database search was complemented by targeted manual searches of reference lists, ESC guidelines, consensus documents, seminal validation studies (e.g., AFEQT), and key implementation papers identified during manuscript development.

### 2.3. Eligibility and Selection Approach

Publications were considered eligible if they addressed at least one of the following areas: (1) AF-specific or generic PROM instruments, including AFEQT, AFSS, EQ-5D, SF-36, or related tools; (2) quality-of-life assessment in AF; (3) implementation of PROMs in clinical practice; (4) healthcare delivery, workflow integration, or outpatient care organization; (5) value-based healthcare or health economic aspects of AF care; (6) digital health, remote monitoring, or electronic PROM collection; or (7) healthcare system or policy implications of patient-reported outcomes.

Original clinical studies, randomized trials, observational studies, registry analyses, validation studies, systematic and narrative reviews, guideline documents, consensus statements, implementation papers, and policy-oriented publications were considered eligible when they provided relevant empirical, methodological, or conceptual information.

Publications were excluded if they were not related to AF, did not address patient-reported outcomes or quality-of-life assessment, focused exclusively on technical electrophysiological outcomes without patient-centered measures, were not available in English, or did not provide sufficient relevance to the healthcare systems or implementation focus of this review.

No formal meta-analysis was performed because of substantial heterogeneity in study design, PROM instruments, patient populations, healthcare settings, implementation models, and reported outcomes. The final selection was therefore intended to support an integrative narrative synthesis rather than a quantitative evidence summary.

The overall literature identification and study selection process is summarized in Figure 1. The review followed an iterative narrative search strategy combining structured database searches with targeted supplementary searches and manual reference screening to identify clinically relevant evidence across implementation, healthcare systems, and patient-reported outcome research.

The literature identification and study selection process are summarized in Figure 1, while the complete database-specific search strategies are presented in Supplementary Table S1.

### 2.4. Narrative Synthesis and Thematic Analysis

The identified literature was analyzed using a thematic narrative synthesis approach. Studies were grouped according to recurring implementation and healthcare system themes, including: symptom and quality-of-life assessment; hidden dimensions of AF burden; longitudinal patient monitoring; healthcare workflow integration; clinician and patient burden; digital infrastructure and interoperability; healthcare utilization and economic implications; implementation challenges in smaller healthcare systems; future directions involving digital health technologies and artificial intelligence-assisted interpretation. Particular attention was given to areas that remain insufficiently represented in conventional AF assessment models, including cognitive symptoms, sleep quality, emotional distress, productivity loss, and social functioning. Rather than focusing exclusively on efficacy outcomes, the review prioritized practical implementation considerations and real-world healthcare delivery challenges that may influence the feasibility and sustainability of PROM integration across diverse clinical environments. The final synthesis included 53 sources covering clinical studies, quality-of-life research, implementation analyses, healthcare policy papers, digital health reports, guideline documents, and broader conceptual frameworks.

### 2.5. Qualitative Assessment of Evidence Strength

Because this study was designed as a narrative implementation review rather than a systematic review or meta-analysis, no formal risk-of-bias assessment tool was applied. The primary objective of the review was to integrate evidence originating from clinically and methodologically heterogeneous sources, including randomized controlled trials, observational studies, PROM validation studies, implementation research, clinical guidelines, consensus documents, health policy publications, and conceptual papers.

To improve transparency, the included literature was interpreted according to an informal qualitative evidence framework. Publications were considered within four broad categories according to their methodological characteristics and intended contribution to the review:

- Category I: High-level empirical evidence, including randomized controlled trials, meta-analyses, systematic reviews, international clinical guidelines, and large multicenter registry studies.
- Category II: Prospective cohort studies, multicenter observational studies, and well-designed validation studies of PROM instruments.
- Category III: Single-center observational studies, retrospective analyses, pilot studies, and descriptive clinical investigations.
- Category IV: Narrative reviews, implementation reports, expert consensus papers, health policy publications, and conceptual articles providing contextual or hypothesis-generating information.

Evidence from higher-level empirical studies primarily informed statements regarding clinical outcomes, patient-reported outcome measures, and healthcare implementation. Lower-level or conceptual publications were used to provide contextual interpretation, describe implementation challenges, identify knowledge gaps, and support discussion of future research directions rather than establish evidence-based clinical conclusions.

Throughout the Results and Discussion, conclusions supported primarily by Category III and Category IV evidence are intentionally presented as exploratory, hypothesis-generating, or conceptual. Conversely, statements regarding clinical outcomes, implementation effectiveness, and patient-centered care are preferentially supported by higher-level empirical evidence (Categories I and II), where available.

### 2.6. Ethical Considerations

This study was based exclusively on previously published literature and did not involve human participants, patient-level data collection, or intervention procedures. Ethical review and informed consent were therefore not required.

### 2.7. Use of Generative Artificial Intelligence

Generative artificial intelligence-assisted tools, specifically ChatGPT, GPT-5.5 Thinking (OpenAI, San Francisco, CA, USA), were used during the linguistic refinement and structural organization of the manuscript, as well as for preliminary conceptual visualization of schematic figures. The scientific content, figure concepts, interpretation of the literature, and final manuscript were critically reviewed, revised, validated, and approved by the authors, who take full responsibility for the scientific integrity and final content of the publication.

## 3. Thematic Findings and Narrative Synthesis

### 3.1. Expanding Interest in Patient-Reported Outcomes in AF Management

Over the past two decades, patient-reported outcome measures (PROMs) have become increasingly incorporated into cardiovascular research and chronic disease management. In atrial fibrillation (AF), this shift has been driven in part by growing recognition that conventional clinical metrics alone do not adequately reflect the complexity of patient experience, prompting development of AF-specific assessment tools [26,27]. Although outcomes such as stroke prevention, hospitalization rates, mortality, and arrhythmia recurrence remain central to AF management, they provide only limited insight into symptom perception, psychological burden, functional status, or day-to-day quality of life.

This discrepancy is particularly relevant in AF because symptom burden frequently indicates poor correlation with objective rhythm findings [28]. Some patients remain minimally symptomatic despite persistent arrhythmia, whereas others experience substantial impairment in physical, emotional, or social functioning even in the absence of sustained AF episodes documented on electrocardiographic monitoring [29]. As a result, increasing attention has been directed toward integrating patient-centered outcome assessment into both clinical studies and routine care pathways.

The growing use of catheter ablation, long-term rhythm monitoring, wearable technologies, and outpatient management programs has further increased interest in PROM-guided evaluation [30]. In many contemporary AF studies, quality-of-life improvement has become an important secondary endpoint alongside conventional cardiovascular outcomes. At the same time, healthcare systems are increasingly expected to demonstrate not only procedural success or complication reduction, but also measurable improvements in patient-perceived well-being and functional recovery.

Nevertheless, the practical role of PROMs in routine AF care remains variably defined. In many institutions, PROM collection continues to function primarily as a research instrument rather than an operational component of longitudinal patient management. Even when PROMs are implemented, interpretation strategies and clinical response pathways often remain inconsistent [31]. Consequently, the expanding use of PROMs has not always translated into systematic integration within real-world healthcare delivery. It should be noted that much of the available evidence supporting PROM use in AF derives from secondary analyses of clinical trials, single-centre observational studies, or registry-based investigations, often conducted in highly resourced academic settings. Prospective multicentre studies specifically designed to evaluate the clinical impact of routine PROM implementation on AF management decisions and patient outcomes remain scarce.

### 3.2. AF-Specific PROM Instruments

Several AF-specific PROM instruments have been developed to improve assessment of symptom burden and disease-related quality of life. Among these, the AFEQT is one of the most widely used tools in both clinical studies and routine practice [12]. The instrument evaluates multiple domains, including symptoms, daily activities, treatment concerns, and treatment satisfaction. Its relatively disease-focused structure has contributed to broad adoption in studies assessing catheter ablation outcomes, rhythm-control strategies, and longitudinal quality-of-life changes [32].

The AFSS represents another commonly referenced AF-specific instrument [13]. Unlike broader quality-of-life questionnaires, the AFSS places greater emphasis on arrhythmia-related symptom severity and episode frequency. This approach may provide practical value in rhythm-monitoring contexts but may also incompletely capture broader psychosocial or functional consequences of AF.

Although the AFEQT and AFSS have undergone formal psychometric validation, much of the evidence supporting their clinical utility derives from controlled research environments, and prospective data demonstrating that routine use of these instruments improves clinical decision-making or patient outcomes in everyday practice remain limited [33]. Moreover, many AF-specific PROMs were initially developed within controlled research environments and may not fully account for the practical constraints of routine outpatient care, where consultation time, staff workload, and digital infrastructure vary substantially.

An additional challenge relates to the dynamic nature of AF itself. Symptom perception may fluctuate over time depending on arrhythmia burden, treatment stage, comorbidities, emotional stress, and patient adaptation. Consequently, single time-point PROM assessment may provide only a partial representation of long-term disease impact [34].

### 3.3. Generic Quality-of-Life Instruments in AF Research

In addition to AF-specific questionnaires, several generic quality-of-life instruments have been widely used in AF populations. The EQ-5D and SF-36 remain among the most frequently applied generic PROMs in cardiovascular research [15,16].

Generic instruments offer several advantages. They facilitate comparison across different chronic diseases, support health economic analyses, and are frequently incorporated into broader value-based healthcare and population health frameworks [35]. In particular, the EQ-5D has become highly relevant in studies involving cost-effectiveness evaluation and quality-adjusted life year (QALY) calculations [36].

However, generic PROMs may demonstrate reduced sensitivity to AF-specific symptom fluctuations. Important arrhythmia-related experiences such as palpitations, irregular pulse awareness, episodic anxiety associated with recurrence, or rhythm-related exercise intolerance may be insufficiently represented in broader quality-of-life domains [37]. As a result, generic instruments may underestimate clinically meaningful changes following rhythm-control interventions or symptom-targeted therapy.

At the same time, broader generic instruments may identify dimensions of disease burden that remain less visible in AF-specific questionnaires, including social isolation, emotional exhaustion, reduced vitality, and functional limitations associated with multimorbidity. This partially explains why many contemporary studies combine disease-specific and generic PROM approaches rather than relying on a single instrument alone.

Existing PROM instruments differ substantially not only in their scope and sensitivity to AF-related symptoms but also in practical implementation characteristics, including respondent burden, administration time, digital adaptability, and potential integration into routine healthcare workflows (Table 1). While AF-specific instruments provide greater sensitivity to rhythm-related symptom changes, generic instruments may offer broader health economic and healthcare system applicability. These implementation characteristics are important considerations when selecting PROMs for routine clinical practice.

The table summarizes the principal AF-specific and generic PROM instruments currently used in clinical practice and research, together with their major strengths, limitations, respondent burden, approximate administration time, digital adaptability, and potential applications within routine atrial fibrillation care. Respondent burden and administration time represent approximate estimates derived from published validation studies and routine clinical implementation and may vary according to the mode of administration. Digital adaptability reflects the feasibility of electronic administration and integration into digital healthcare platforms rather than formal regulatory certification. AF-specific tools may better capture rhythm-related symptom burden, whereas generic instruments may provide broader insight into psychosocial functioning and healthcare economic implications.

### 3.4. PROMs Within Contemporary Healthcare Models

The increasing incorporation of PROMs into AF care reflects broader transitions occurring across healthcare systems internationally. Contemporary healthcare models increasingly emphasize patient-centered care, long-term disease management, shared decision-making, and value-oriented outcome assessment [18,19]. Within this context, PROMs are increasingly viewed not only as research endpoints, but also as potential indicators of healthcare quality and longitudinal treatment effectiveness.

In some healthcare systems, PROM collection has already been integrated into registry programs, digital outpatient platforms, and post-procedural follow-up pathways [38]. Remote monitoring ecosystems and electronic questionnaire platforms have further expanded the feasibility of repeated PROM assessment outside traditional clinic visits. These developments became particularly relevant following the expansion of telemedicine and remote cardiovascular care during and after the COVID-19 pandemic [39]. However, the evidence base for these developments remains largely descriptive. Most published reports on PROM integration into digital AF care platforms are based on feasibility assessments, pilot programs, or institutional case studies rather than comparative effectiveness studies with patient-level outcome data.

Despite these advances, implementation remains highly uneven across healthcare environments. Large academic centers and digitally integrated healthcare systems may possess the infrastructure required for systematic PROM collection and interpretation, whereas smaller institutions often face substantial logistical barriers. Even in technologically advanced settings, the practical clinical significance of repeated PROM collection remains debated when actionable interpretation strategies are poorly defined [40].

Importantly, increasing questionnaire volume may also contribute to patient fatigue and reduced long-term adherence. In routine clinical environments, PROM implementation competes with multiple parallel documentation requirements, electronic reporting systems, and administrative obligations. As a result, successful integration may depend less on the number of collected metrics and more on whether PROM results meaningfully influence clinical decision-making and patient management.

### 3.5. Hidden Dimensions of AF Burden Underrepresented in Current PROM Frame Works

Although AF management has become increasingly sophisticated, routine assessment still tends to prioritize rhythm status, stroke prevention, symptom control, and major cardiovascular events. These endpoints are essential, but they do not fully capture how patients experience AF over months or years. In everyday practice, some of the most disabling aspects of AF are not always the most visible during a cardiology consultation.

This limitation is partly methodological. Many PROM instruments used in AF were developed to evaluate symptoms, functional limitation, treatment satisfaction, or general health status. These domains are important, but they may not fully represent the broader burden of living with a chronic, recurrent, and often unpredictable arrhythmia. Patients may describe fatigue, fear of recurrence, poor sleep, concentration problems, or reduced confidence in daily activities even when conventional clinical indicators appear relatively stable [41,42].

The issue is not that existing PROMs are irrelevant. Rather, their current use may be too narrow. When PROMs are treated mainly as symptom scores, their ability to reveal broader health system needs becomes limited. In this sense, underrecognized domains of AF burden are not only clinical blind spots; they may also represent missed opportunities for earlier intervention, more appropriate follow-up, and better resource allocation.

#### 3.5.1. Cognitive Symptoms and Perceived Mental Performance

Cognitive dysfunction has received increasing attention in AF research, particularly because AF is associated with stroke, silent cerebral infarction, cerebral hypoperfusion, vascular disease, and dementia risk [43,44]. However, the cognitive burden experienced by patients is not limited to formally diagnosed cognitive impairment. Many patients describe more subtle problems, including reduced concentration, slower thinking, memory concerns, or mental fatigue. These symptoms may affect medication adherence, self-monitoring, work capacity, and the ability to participate in shared decision-making.

Several observational studies have reported an association between AF and cognitive impairment beyond overt stroke. For example, the Swiss-AF cohort (prospective multicentre cohort; >2400 participants) demonstrated that cognitive dysfunction was common even among anticoagulated patients and was associated with a higher overall disease burden [41,45]. However, the observational design precludes causal inference, and cognitive decline cannot be attributed exclusively to AF itself because of potential confounding by age, vascular comorbidity, and multimorbidity.

Current PROM strategies in AF rarely assess cognitive symptoms in a systematic way. Generic quality-of-life tools may capture some aspects of mental functioning, but they are usually not sensitive enough to distinguish cognitive complaints from emotional distress, fatigue, or general health perception. AF-specific instruments, in turn, often focus more strongly on palpitations, dyspnea, activity limitation, and treatment concerns.

This gap may be clinically relevant. A patient who reports poor concentration, reduced confidence, or difficulty managing daily routines may require a different follow-up strategy than a patient whose symptoms are limited to occasional palpitations. In older patients or those with multimorbidity, cognitive complaints may also influence anticoagulation adherence, fall risk discussion, treatment preferences, and caregiver involvement. For healthcare systems, failure to recognize this domain may contribute to avoidable complications or inefficient follow-up.

Overall, evidence linking AF with cognitive dysfunction is supported by multiple prospective cohort studies and registry analyses; however, the specific contribution of PROM-based cognitive assessment to routine AF management remains insufficiently investigated.

#### 3.5.2. Sleep Disturbance and Fatigue

Sleep disturbance is another important but often insufficiently characterized dimension of AF burden. AF episodes may occur at night, cause palpitations or anxiety, and lead to fragmented sleep. Conversely, sleep disorders, particularly obstructive sleep apnea, are strongly associated with AF development, recurrence, and treatment resistance [46,47]. This bidirectional relationship makes sleep highly relevant both as a symptom domain and as a modifiable clinical factor.

In the CAPTAF randomized trial (155 patients), catheter ablation resulted in greater improvements in patient-reported quality of life than antiarrhythmic drug therapy, with fatigue representing one of the domains contributing to overall health status improvement [48]. Nevertheless, fatigue was not evaluated as an independent primary endpoint, limiting conclusions regarding AF-specific fatigue mechanisms.

Fatigue is frequently reported by AF patients, yet it remains difficult to interpret. It may reflect arrhythmia burden, rate control medication, heart failure, sleep disruption, anxiety, deconditioning, anemia, thyroid dysfunction, or other comorbidities. In routine care, fatigue is sometimes treated as a nonspecific complaint rather than a structured outcome requiring longitudinal assessment.

Existing PROMs may capture fatigue only partially. AFEQT includes activity-related and symptom-related domains, while generic tools such as SF-36 include vitality-related items. However, these instruments may not adequately differentiate between daytime sleepiness, exercise intolerance, emotional exhaustion, medication-related tiredness, and AF-related fatigue. For implementation purposes, this distinction matters. A low score caused by sleep apnea requires a different response than a low score caused by poor rate control or post-procedural anxiety.

Current evidence suggests that sleep disturbance and fatigue represent clinically relevant components of AF burden; however, available studies remain heterogeneous regarding assessment methods and outcome definitions.

A more refined approach to sleep and fatigue assessment may therefore support more targeted AF management. It may also help identify patients who need sleep studies, medication review, rehabilitation, psychological support, or closer rhythm monitoring.

#### 3.5.3. Emotional Distress, Uncertainty, and Fear of Recurrence

AF is not only a rhythm disorder; for many patients, it is also a condition marked by unpredictability. Episodes may occur suddenly, symptoms may fluctuate, and patients may struggle to understand when palpitations are dangerous and when they are not. This uncertainty can create persistent psychological distress, particularly in patients with recurrent symptomatic episodes or previous emergency department visits [49].

Representative evidence of several observational studies have consistently demonstrated an association between anxiety, depressive symptoms, and reduced health-related quality of life in AF populations. For example, Gehi et al. (prospective observational study; *n* = 150) reported that psychological distress was independently associated with greater symptom burden and poorer patient-reported health status [50]. Likewise, Son et al. [51] summarized evidence from multiple observational studies demonstrating that emotional well-being is a major determinant of perceived quality of life in AF. However, most available evidence remains observational or cross-sectional, limiting causal inference regarding the relationship between emotional distress and AF progression.

Several observational studies using AFEQT and generic quality-of-life instruments consistently reported associations between anxiety, depressive symptoms, and reduced quality of life in AF populations. However, most available studies are cross-sectional, making it difficult to determine causal relationships between emotional distress and AF progression.

Anxiety and depressive symptoms are common in AF populations and have been associated with worse quality of life, higher symptom reporting, and increased healthcare utilization [51,52]. However, emotional distress in AF is not always equivalent to a psychiatric disorder. Some patients experience situational anxiety driven by fear of recurrence, fear of stroke, uncertainty about anticoagulation, or lack of confidence after cardioversion or ablation.

PROMs can help identify this emotional burden, but current implementation is inconsistent. If psychological distress is measured but not linked to any clinical action, the value of measurement becomes questionable. In a busy outpatient clinic, documenting anxiety without creating a pathway for education, reassurance, medication review, rhythm monitoring, or psychological referral may simply add another data point without improving care.

This is where a healthcare system perspective becomes important. Emotional distress should not be viewed only as an individual patient characteristic. It may also reflect gaps in communication, fragmented follow-up, limited patient education, and poor continuity after acute AF episodes.

The association between AF and emotional distress is consistently demonstrated across observational studies and validated PROM investigations, although prospective intervention studies evaluating PROM-guided management remain limited.

#### 3.5.4. Sexual Health and Intimacy

Sexual health remains one of the least discussed dimensions of AF-related quality of life. Patients may experience reduced sexual activity or intimacy because of fear of triggering arrhythmia, reduced exercise tolerance, medication effects, comorbid cardiovascular disease, anxiety, or body-image concerns after procedures. Yet, this domain is rarely assessed systematically in AF care.

Although evidence remains limited, several quality-of-life studies have identified sexual functioning as an underrecognized determinant of patient well-being following AF diagnosis and treatment. Existing evidence is derived predominantly from observational studies and secondary analyses rather than dedicated prospective investigations, highlighting an important gap in current PROM development.

The omission is understandable but problematic. Sexual health is often underreported by patients and underexplored by clinicians, particularly when consultations are time-limited or focused on anticoagulation, rhythm strategy, and procedural planning. Some patients may not raise the issue unless directly invited to do so. Others may assume that sexual concerns are not relevant to cardiology care.

Most used AF PROMs do not evaluate sexual health in sufficient detail. This may lead to an overly narrow understanding of functional recovery after treatment. A patient may suggest improvement in rhythm control and general symptom burden but continue to experience reduced confidence, avoidance behavior, or relationship strain. From a patient-centered care perspective, this represents an important residual burden.

In contrast, AF-specific evidence regarding sexual health remains limited, and current knowledge is derived predominantly from small observational studies and extrapolation from broader cardiovascular populations.

In future PROM development or adaptation, sexual health does not necessarily require extensive questionnaire modules. Even brief, optional, culturally sensitive items may help identify patients who need counseling, medication review, or reassurance. This is particularly relevant if AF care is expected to move beyond survival and event prevention toward broader restoration of daily life.

#### 3.5.5. Social Participation, Work Productivity, and Caregiver Burden

AF may affect social participation and productivity in ways that are not always captured by clinical endpoints. Patients may avoid travel, physical activity, social events, or work responsibilities because of unpredictable symptoms or fear of acute recurrence. Those with frequent episodes may require repeated medical visits, emergency assessments, medication changes, or procedures, each of which can generate indirect costs for patients, families, and employers [53].

The ORBIT-AF registry (prospective multicentre observational registry; *n* ≈ 10,000) demonstrated that greater AF symptom burden was associated with poorer patient-reported health status and reduced functional status [5,51]. However, the registry was not specifically designed to evaluate work productivity or caregiver burden. Subsequent systematic reviews suggest that these domains remain insufficiently represented within currently available AF-specific PROM instruments.

Work-related impairment is especially important in younger or working-age AF populations. Absenteeism and presenteeism may occur even when patients are not hospitalized. For healthcare systems, these indirect consequences are often less visible than procedural costs or admission rates, but they are highly relevant to value-based care and health economic evaluation.

Caregiver burden is another underrecognized area. Family members may assist with medication management, transport to appointments, monitoring of symptoms, or decision-making during acute episodes. In older patients, particularly those with cognitive impairment or multimorbidity, caregiver involvement may become essential for safe long-term management. Yet, caregiver burden is rarely integrated into routine AF outcome assessment.

Evidence specifically addressing caregiver burden in AF remains scarce. Most available data originate from qualitative research and chronic disease literature rather than prospective AF-specific investigations. Although impaired work productivity has been reported in several observational cohorts, the overall evidence base remains limited, and further prospective studies are required to quantify the socioeconomic impact of AF more comprehensively.

A more complete PROM strategy should therefore consider not only the patient’s symptoms, but also the patient’s capacity to function socially, professionally, and within the family environment. This does not mean that every AF clinic must administer long social or economic questionnaires. Rather, it suggests that PROM implementation should be designed with enough flexibility to identify when these domains are clinically relevant.

#### 3.5.6. Implications for PROM Development and Implementation

**Figure 2:**
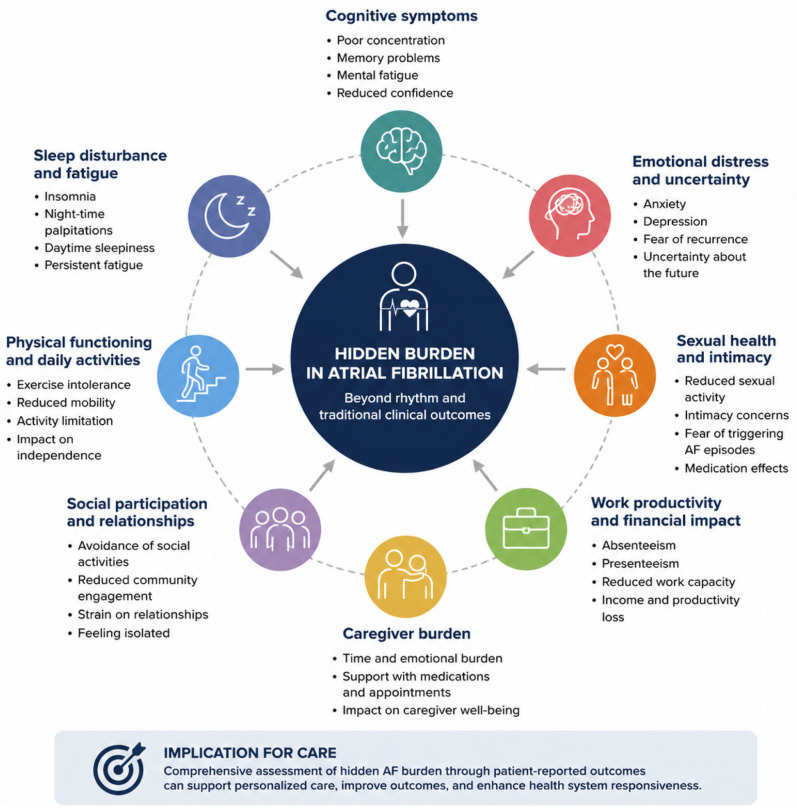
Hidden dimensions of atrial fibrillation burden extending beyond conventional symptom assessment. The figure illustrates the multidimensional concept of the hidden burden experienced by patients with atrial fibrillation. It is intended as a conceptual framework and does not imply equivalent levels of evidence or clinical importance across all domains. As discussed in the text, cognitive dysfunction, emotional distress, and sleep disturbance are supported by comparatively stronger AF-specific evidence, whereas sexual health, caregiver burden, and productivity loss remain comparatively underexplored and require further prospective investigation. Colors are used solely to distinguish conceptual domains and do not indicate relative importance or strength of evidence. Arrows represent conceptual relationships between the hidden burden of atrial fibrillation and its multidimensional clinical manifestations rather than causal pathways.

The hidden dimensions described above suggest that AF-related burden is broader than symptom frequency or arrhythmia-related discomfort alone (Figure 2). Cognitive symptoms, sleep disturbance, emotional distress, sexual health, productivity loss, and caregiver burden may each influence treatment satisfaction, adherence, healthcare utilization, and long-term quality of life.

For future implementation, two points are particularly important. First, PROM selection should depend on the intended use. A short symptom-focused tool may be appropriate for rapid outpatient screening, whereas a broader multidomain assessment may be more useful after cardioversion, ablation, recurrent hospitalization, or treatment failure. Second, PROM results must be linked to clinical or organizational action. Otherwise, even well-designed questionnaires risk becoming administrative exercises rather than instruments of patient-centered care.

This distinction is central to the present review. The purpose of PROM integration should not be to collect more data for its own sake. Its value lies in whether patient-reported information can help clinicians and healthcare systems recognize unmet needs, adapt follow-up intensity, and improve care delivery in a way that is feasible in real-world settings.

Figure 2 provides a conceptual overview of the multidimensional hidden burden of AF rather than a hierarchy of evidence supporting individual domains.

Collectively, the evidence reviewed demonstrates that the hidden burden of atrial fibrillation is multidimensional; however, the robustness of supporting evidence differs substantially across individual domains. Cognitive dysfunction, emotional well-being, and sleep disturbance are supported by comparatively stronger AF-specific evidence, whereas sexual health, caregiver burden, and productivity loss remain relatively underexplored and are supported primarily by smaller observational studies or extrapolation from broader cardiovascular and chronic disease literature. Consequently, these latter domains should currently be regarded as important emerging areas for future investigation rather than routinely established components of comprehensive AF assessment.

### 3.6. Current and Future Operational Applications of PROMs in Atrial Fibrillation Care

#### 3.6.1. From Symptom Assessment to Longitudinal Patient Monitoring

Historically, PROMs in AF care have been used primarily to quantify symptom burden and evaluate treatment-related changes in quality of life. In many studies, PROMs function mainly as secondary endpoints used to compare rhythm-control strategies, catheter ablation outcomes, or pharmacological interventions [48,54]. While this role remains important, such an approach may underestimate the broader operational value of patient-reported information within long-term healthcare delivery.

AF is a chronic and dynamic condition characterized by fluctuations in symptoms, treatment response, emotional adaptation, and healthcare utilization over time. As a result, isolated clinical encounters may provide only a limited representation of overall disease trajectory. Representative evidence supporting longitudinal PROM monitoring remains limited. Most available data originate from integrated care programs such as the nurse-led AF management trial by Hendriks et al. [55], which demonstrated improved guideline adherence and clinical outcomes through structured longitudinal follow-up rather than PROM-guided monitoring alone. Consequently, extrapolation to routine PROM-based monitoring should be interpreted cautiously.

Repeated PROM assessment has the potential to detect gradual deterioration that may not be immediately apparent through conventional rhythm monitoring alone; however, this hypothesis has not yet been confirmed in prospective AF-specific implementation studies. For example, worsening fatigue, declining physical function, increasing anxiety, or reduced confidence in daily activities may precede hospitalization, treatment discontinuation, or recurrent emergency department utilization [55]. Available evidence suggests that repeated PROM assessment may facilitate earlier recognition of symptom progression and functional decline. However, this potential role remains supported primarily by observational evidence and integrated care programs rather than prospective AF-specific implementation trials. No randomized studies have yet demonstrated that longitudinal PROM monitoring independently improves clinical outcomes compared with standard follow-up.

Importantly, longitudinal PROM monitoring does not necessarily require highly complex infrastructure. Even relatively simple repeated assessments performed at predefined intervals may provide additional clinical context during outpatient management. However, the usefulness of longitudinal PROM collection depends largely on whether reported changes trigger meaningful clinical interpretation and action. PROMs that are collected but not operationalized risk becoming administrative rather than clinical tools.

#### 3.6.2. PROMs and Outpatient Care Optimization

Healthcare systems managing large AF populations increasingly face pressure related to outpatient workload, repeated follow-up visits, and growing demand for chronic disease monitoring. In this setting, PROMs may contribute to more individualized and resource-sensitive care organization.

The mAFA-II program demonstrated that digitally supported integrated AF management can improve adherence to guideline-directed care and reduce adverse clinical outcomes [56]. Nevertheless, PROM-guided outpatient prioritization itself was not evaluated as an independent intervention, and therefore direct evidence supporting PROM-based triage remains limited.

Not all patients with AF require the same intensity of monitoring. Some individuals remain clinically stable for prolonged periods with minimal symptoms and preserved functional status, whereas others experience fluctuating symptom burden, recurrent anxiety, impaired treatment adherence, or repeated healthcare utilization despite apparently acceptable rhythm control.

PROM-guided outpatient prioritization therefore represents a promising implementation strategy rather than an established evidence-based component of routine AF care. Future prospective studies should determine whether PROM-guided triage improves healthcare efficiency, resource allocation, or patient outcomes.

PROM-informed outpatient management may also improve communication during consultations. Structured patient-reported data can help clinicians focus discussions on domains that are most relevant to the individual patient rather than relying exclusively on standard rhythm-focused questioning. This may be particularly important in AF, where symptom burden is often multifactorial and influenced by comorbidities, psychological distress, sleep disorders, and social circumstances.

Nevertheless, outpatient implementation remains challenging. In busy clinical settings, additional questionnaires may be perceived as another administrative requirement competing with already substantial documentation obligations. If PROM collection increases workload without clearly influencing clinical decision-making, long-term adherence by both clinicians and patients may decline. Successful implementation therefore depends not only on data collection itself, but also on workflow integration and practical interpretability.

#### 3.6.3. PROMs as Healthcare Quality Indicators

Beyond individual patient management, PROMs are increasingly discussed as potential indicators of healthcare quality and treatment effectiveness. The American Heart Association Scientific Statement on patient-reported health status and subsequent implementation initiatives have emphasized the importance of incorporating patient-reported outcomes into cardiovascular quality assessment. However, these recommendations are based predominantly on implementation experience and expert consensus rather than prospective AF-specific comparative studies. Although increasingly discussed within healthcare policy and implementation literature, this application has not yet been validated by large prospective AF-specific implementation studies. Traditional cardiovascular quality metrics often focus on mortality, complications, readmissions, procedural outcomes, or guideline adherence [17]. While these measures remain essential, they may incompletely capture whether patients experience meaningful improvement in daily functioning and quality of life.

This issue is particularly relevant in AF because symptom relief and functional recovery are often central therapeutic goals. A technically successful intervention does not necessarily translate into perceived improvement from the patient perspective. Conversely, some patients report substantial improvement despite incomplete rhythm normalization or persistent intermittent arrhythmia episodes.

As healthcare systems increasingly emphasize patient-centered care, PROMs may provide complementary information regarding the real-world impact of treatment strategies. Several studies have suggested that patient-reported quality-of-life improvement following catheter ablation may correlate more strongly with treatment satisfaction than arrhythmia recurrence rates alone [57]. Similar considerations apply to rate-control strategies, anticoagulation management, and integrated outpatient care programs.

PROMs are increasingly discussed as complementary indicators of healthcare quality because they capture dimensions of treatment benefit that conventional cardiovascular performance metrics cannot fully reflect. Nevertheless, current evidence supporting this application derives predominantly from implementation studies, registry analyses, and expert consensus rather than prospective AF-specific comparative trials. Consequently, PROMs should currently be regarded as complementary rather than standalone healthcare quality indicators.

#### 3.6.4. PROMs Within Value-Based Healthcare Models

PROM integration is closely aligned with the principles of value-based healthcare (VBHC), where outcomes meaningful to patients are considered alongside healthcare resource utilization. However, evidence supporting PROM-guided value assessment in AF remains largely conceptual and policy-oriented. Prospective studies demonstrating measurable improvements in healthcare efficiency, resource allocation, or reimbursement outcomes are currently lacking [18,58,59].

AF represents an important condition within this discussion because of its long-term management requirements and recurrent healthcare interactions. Hospitalizations, emergency visits, repeat procedures, anticoagulation monitoring, and outpatient reassessment contribute substantially to healthcare expenditures associated with AF [60]. At the same time, many AF-related consequences—including productivity loss, social limitation, reduced exercise capacity, and emotional burden—remain difficult to quantify using conventional biomedical indicators alone. The conceptual alignment between PROMs and VBHC frameworks is well established in the broader health policy literature [18,61]. However, empirical evidence demonstrating that PROM-informed value assessment changes resource allocation decisions, reimbursement pathways, or measurable healthcare outcomes in AF populations is currently absent. Most existing applications of PROMs within VBHC remain at the level of conceptual frameworks, institutional pilot programs, or registry-based quality reporting rather than prospective outcome evaluations.

PROMs may therefore help expand evaluation beyond traditional event-based metrics. In some settings, patient-reported outcomes are already being incorporated into value-oriented care models, integrated care pathways, and digital chronic disease management programs [61]. This may become increasingly relevant as healthcare systems seek approaches capable of balancing cost containment with preservation of patient-centered care quality.

Nevertheless, implementation within VBHC models should be approached cautiously. There is a risk that PROM collection may become excessively metric-driven, emphasizing numerical scoring over meaningful clinical interpretation. Overstandardization may also reduce flexibility needed for individualized care. In addition, healthcare systems with limited digital infrastructure or workforce capacity may struggle to implement repeated PROM collection at scale without creating additional administrative burden.

As a result, successful integration into value-based care likely requires selective and clinically purposeful PROM use rather than indiscriminate expansion of questionnaire-based assessment.

The potential role of PROMs in AF management extends beyond symptom quantification alone. Depending on implementation strategy and healthcare infrastructure, PROM-guided assessment may contribute to longitudinal patient monitoring, outpatient care prioritization, healthcare quality evaluation, and value-based care initiatives. However, the practical utility of PROM integration depends substantially on workflow feasibility, interpretability, and organizational support. Key potential operational applications of PROMs in AF healthcare delivery are summarized in Table 2.

Table 2: Current and potential operational applications of patient-reported outcome measures (PROMs) in atrial fibrillation care. The table summarizes the principal operational applications of PROMs identified in the present narrative implementation review together with their potential impact on clinical practice and healthcare systems. The Evidence Status column represents a qualitative interpretation of the current literature and is not intended as formal evidence-grading system. Evidence-supported indicates applications supported by consistent evidence from clinical studies, registries, or guideline recommendations. Emerging evidence refers to applications supported by preliminary observational or implementation studies but requiring further prospective validation. Future implementation opportunity denotes conceptual or implementation-oriented applications that are biologically and clinically plausible but currently lack robust AF-specific prospective evidence.

**Figure 3:**
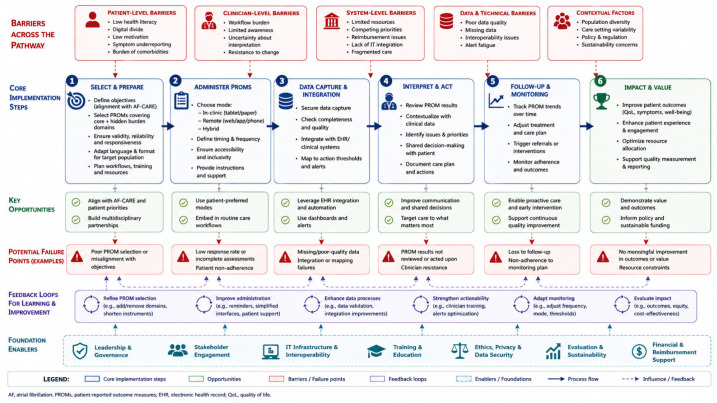
Conceptual Framework for PROM Implementation in Atrial Fibrillation Care: Opportunities, Barriers, and Continuous Feedback. Color coding distinguishes the major conceptual components of the framework (core implementation steps, opportunities, barriers/failure points, feedback loops, and foundational enablers), while solid arrows indicate the implementation process flow and dashed arrows represent feedback and continuous learning pathways, as detailed in the figure legend.

The integration of PROMs into AF management may be conceptualized as a dynamic and iterative process extending across the broader healthcare delivery continuum. Rather than functioning solely as isolated questionnaires, PROMs may contribute to longitudinal monitoring, clinical interpretation, care adaptation, and healthcare quality evaluation when integrated into sustainable organizational and digital infrastructures. A conceptual framework for PROM integration across AF healthcare delivery is presented in Figure 3.

Figure 3 illustrates a conceptual implementation framework describing how patient-reported outcome measures (PROMs) may be integrated into routine atrial fibrillation care. In contrast to a purely linear implementation pathway, the framework incorporates real-world implementation barriers—including patient non-adherence, clinician resistance, workflow disruption, resource limitations, digital interoperability, and data quality issues—that may influence successful PROM integration at multiple stages. Continuous evaluation and feedback are emphasized as essential components of sustainable implementation, supporting ongoing refinement of PROM collection, interpretation, and integration into patient-centred AF-CARE. PROM data may support longitudinal monitoring, clinical interpretation, care adaptation, and healthcare outcome evaluation within patient-centered and value-based healthcare models. Sustainable implementation depends on workflow integration, digital infrastructure, multidisciplinary collaboration, patient engagement, and organizational support. Accordingly, these applications should currently be regarded as promising implementation concepts rather than evidence-based standards of routine AF care.

#### 3.6.5. Limitations of PROM Operationalization in Real-World Practice

Although PROMs offer important opportunities to strengthen patient-centred AF management, successful operational implementation depends on considerably more than questionnaire selection alone. The findings of the present review indicate that several practical and organizational factors continue to limit the routine integration of PROMs across diverse healthcare environments.

One important challenge relates to questionnaire burden. Patients with AF are frequently older, multimorbid, and managed across multiple specialties, resulting in repeated requests to complete health-related questionnaires. Excessively long or frequent PROM assessments may reduce patient engagement and compromise the completeness and quality of collected data. Consequently, implementation strategies should prioritize concise, clinically relevant instruments and adaptive assessment schedules whenever possible.

A second limitation concerns interpretation. PROM scores should not be viewed as isolated numerical indicators because changes may reflect a combination of arrhythmia burden, treatment effects, comorbid conditions, emotional well-being, and broader psychosocial circumstances. Their clinical value therefore depends on interpretation within the overall clinical context and on the availability of clearly defined response pathways.

Operational feasibility also varies substantially between healthcare systems. Institutions with integrated electronic health records, digital PROM platforms, and multidisciplinary care models are generally better positioned to implement longitudinal PROM assessment than healthcare environments relying on fragmented documentation systems or limited digital infrastructure. Consequently, implementation strategies should remain sufficiently flexible to accommodate differences in organizational capacity, workforce resources, and local healthcare priorities.

Taken together, these observations suggest that PROM implementation should be regarded as an organizational process rather than simply the introduction of additional questionnaires. The broader healthcare applications discussed throughout this review—including longitudinal monitoring, outpatient prioritization, healthcare quality assessment, and value-based care—should therefore be interpreted as promising implementation concepts supported by varying levels of evidence rather than as established standards of routine AF management. Future prospective implementation studies are needed to determine how PROM-guided care can be integrated into routine clinical practice while improving patient-centred outcomes, workflow efficiency, and healthcare system performance.

The 2024 ESC Guidelines emphasize the AF-CARE pathway as the principal framework for comprehensive atrial fibrillation management. Although PROMs are increasingly recognized as important components of patient-centred care, their contribution differs across individual AF-CARE domains and is supported by varying levels of empirical evidence. Table 3 summarizes the current evidence regarding the potential role of PROMs within each component of the AF-CARE pathway together with the principal knowledge gaps identified in the present review.

To provide a more systematic overview of the relationship between PROM domains and the contemporary AF-CARE framework, the principal applications of PROMs across each AF-CARE component together with the current strength of evidence and remaining knowledge gaps are summarized in Table 3.

Table 3 summarizes the potential contribution of PROMs within each component of the AF-CARE framework, together with representative supporting evidence, the current level of evidence, and the principal knowledge gaps. Although PROMs are well-established for measuring symptom burden and health-related quality of life, several broader healthcare system applications—including longitudinal monitoring, quality assessment, and value-based care—remain supported primarily by observational studies, implementation initiatives, and conceptual frameworks, highlighting the need for prospective AF-specific implementation research.

## 4. Discussion

### 4.1. Reframing PROMs in AF-CARE

The present review suggests that the role of patient-reported outcome measures (PROMs) in atrial fibrillation (AF) management may extend considerably beyond conventional symptom assessment alone. Historically, PROMs in AF have largely been viewed as supplementary research instruments designed to evaluate treatment response or quality-of-life changes following rhythm-control interventions [12,13,48,54]. However, the increasing complexity of chronic AF management, together with growing emphasis on patient-centered and value-based care, has created a broader context in which PROMs may also function as operational healthcare tools [18,19,61].

Throughout this review, proposed healthcare system applications of PROMs should be interpreted within the context of the currently available evidence. While their value for assessing symptom burden and health-related quality of life is well established, broader implementation roles—including healthcare quality assessment, outpatient prioritization, and value-based care—remain supported primarily by observational evidence, implementation studies, and conceptual healthcare frameworks rather than prospective comparative trials.

This shift is important because conventional AF assessment remains heavily focused on rhythm status, procedural outcomes, hospitalization rates, and cardiovascular complications [2,4]. Although these parameters remain clinically essential, they do not consistently reflect the lived experience of patients with AF [9,37]. The findings of the present review suggest that important dimensions of disease burden—including cognitive symptoms, emotional distress, sleep disturbance, reduced social participation, and productivity impairment—may remain insufficiently represented within traditional cardiovascular evaluation models [41,42,43,44,46,47,49,51,52].

Importantly, this does not imply that PROMs should replace conventional clinical assessment. Rather, PROM-guided evaluation may complement biomedical metrics by providing additional insight into how AF influences patient functioning over time. This distinction is particularly relevant in chronic disease management, where long-term adaptation, treatment expectations, and symptom perception may evolve independently from objective rhythm findings [28,29].

The broader operational potential of PROMs also deserves attention. Repeated patient-reported assessment may help support longitudinal monitoring, outpatient prioritization, healthcare quality evaluation, and multidisciplinary care coordination [20,55,62]. In this sense, PROMs may contribute not only to individual patient management, but also to healthcare system responsiveness and resource-sensitive care delivery. Nevertheless, successful implementation requires realistic integration into routine clinical workflows rather than isolated questionnaire collection detached from clinical action [23,40].

### 4.2. Why Real-World Implementation Remains Difficult

Despite growing interest in PROM-guided AF care, implementation remains considerably more challenging than conceptual frameworks often suggest. The evidence reviewed in this study indicates that successful PROM integration depends not only on questionnaire availability but also on workflow feasibility, digital infrastructure, clinician engagement, organizational support, and sustainable interpretation pathways. Importantly, the implementation barriers summarized in Table 4 should not be viewed as isolated technical problems but as interconnected organizational challenges that influence the long-term sustainability of PROM-guided care.

One of the principal barriers relates to clinical workflow. Contemporary AF management already requires extensive documentation, rhythm assessment, anticoagulation management, and multidisciplinary coordination [39]. Consequently, PROM collection may be perceived as an additional administrative task unless patient-reported information directly informs clinical decision-making. Similarly, patient adherence may decline when questionnaires are lengthy, repetitive, or insufficiently integrated into routine care [20,23,62].

Interpretation of PROM data represents an additional challenge. Changes in patient-reported outcomes may reflect arrhythmia burden, emotional distress, comorbidity progression, medication effects, or broader psychosocial circumstances [21,49,52]. Accordingly, PROM scores should always be interpreted within the broader clinical context rather than used as isolated numerical indicators.

Digital infrastructure also substantially influences implementation feasibility [23,24]. Healthcare systems with interoperable electronic health records and integrated remote monitoring platforms are generally better positioned to incorporate PROMs into routine care than institutions relying on fragmented documentation systems [62]. Consequently, implementation strategies should remain adaptable to local organizational capacity rather than assuming universal digital readiness [40].

Overall, the present review suggests that PROM implementation should be regarded as an organizational process rather than simply the introduction of new questionnaires. Sustainable integration is likely to depend on clinically meaningful interpretation, multidisciplinary collaboration, and workflow designs that translate patient-reported information into actionable care pathways. Key implementation challenges and potential mitigation strategies are summarized in Table 4.

Table 4: Major barriers that may complicate implementation of patient-reported outcome measures (PROMs) in atrial fibrillation (AF) care. Successful integration depends not only on questionnaire selection, but also on workflow feasibility, digital infrastructure, interpretability, organizational support, and patient engagement within diverse healthcare environments.

The implementation barriers identified in the present review are broadly consistent with established implementation science frameworks, including the Consolidated Framework for Implementation Research (CFIR) [63], the RE-AIM framework [64], and Normalization Process Theory (NPT) [65]. These frameworks emphasize that successful implementation depends not only on the intervention itself but also on organizational context, stakeholder engagement, workflow integration, leadership support, and continuous evaluation [63,64,65]. Although the present narrative review was not explicitly structured around a single implementation science framework, many of the implementation challenges identified—including clinician engagement, digital interoperability, resource availability, patient adherence, and integration into routine workflows—closely correspond to constructs described within these established models [63,64,65]. Future implementation studies evaluating PROM-guided AF care may benefit from the systematic application of implementation science frameworks to facilitate reproducibility, scalability, and sustainable integration across diverse healthcare settings [63,64,65]. For example, clinician engagement and organizational readiness correspond closely to CFIR constructs, healthcare reach and sustainability are reflected within the RE-AIM framework, whereas long-term integration into routine clinical workflows is central to Normalization Process Theory [63,64,65].

### 4.3. Challenges in Smaller and Underrepresented Healthcare Systems

Many discussions regarding PROM implementation originate from highly specialized academic centers or healthcare systems with substantial digital infrastructure and organizational capacity. However, implementation feasibility may differ considerably in smaller or underrepresented healthcare environments. Consequently, the transferability of these findings to smaller or resource-constrained healthcare environments should be interpreted cautiously until further prospective implementation studies become available.

Several challenges appear particularly relevant in such settings. Fragmented continuity of care, workforce limitations, restricted access to multidisciplinary services, and variable digital integration may complicate systematic PROM implementation operationally [25]. In some healthcare systems, AF management remains distributed across multiple providers without centralized longitudinal coordination, making repeated patient-reported assessment difficult to standardize operationally.

Language adaptation and cultural interpretation also remain important considerations. PROM instruments validated in one healthcare environment may not fully reflect patient priorities, communication patterns, or health perception in another [31]. Even when validated translations exist, implementation strategies may require local adaptation to remain clinically meaningful and feasible.

Importantly, smaller healthcare systems may simultaneously possess unique advantages. More compact organizational structures may facilitate faster implementation of integrated outpatient pathways or multidisciplinary collaboration once institutional support is established. In addition, increasing use of telemedicine and digital monitoring technologies may gradually reduce some traditional infrastructure limitations [39].

Nevertheless, the present review suggests that PROM implementation strategies should not be viewed as universally transferable between healthcare systems. Flexible adaptation to local organizational realities is likely necessary for sustainable long-term integration.

### 4.4. Digital Health as an Enabler Rather than a Standalone Solution

Digital health technologies have emerged as one of the most promising facilitators of PROM implementation in atrial fibrillation care, offering opportunities for automated data collection, remote symptom monitoring, integration with wearable devices, and more continuous assessment of patient-reported outcomes [17,18,57,60]. Digital platforms may also reduce administrative burden, improve longitudinal data capture, and facilitate communication between patients and multidisciplinary healthcare teams.

Artificial intelligence may ultimately support the interpretation of longitudinal PROM trajectories, integration with wearable data, and individualized follow-up strategies. However, evidence supporting AI-assisted PROM implementation in atrial fibrillation currently remains limited to early digital health initiatives and conceptual frameworks. Consequently, AI should presently be regarded as a promising future direction rather than an established component of routine AF management.

Nevertheless, the present review suggests that digitalization alone is insufficient to ensure successful PROM implementation [39]. Most published evidence originates from feasibility studies, pilot implementation programmes, or integrated care initiatives, whereas robust prospective studies evaluating the independent impact of digital PROM integration on clinical outcomes, healthcare efficiency, or resource utilization remain limited [17,57,60]. Furthermore, healthcare systems differ substantially in digital maturity, interoperability of electronic health records, and organizational readiness, making universal implementation strategies difficult to generalize [25,39].

Artificial intelligence and automated clinical decision-support systems may further enhance the interpretation of longitudinal PROM data by identifying clinically meaningful symptom trajectories and supporting individualized follow-up strategies. However, these applications remain largely exploratory within AF care and require prospective validation before routine clinical adoption can be recommended [60].

Consequently, digital health should be regarded primarily as an enabling infrastructure that facilitates PROM implementation rather than as an intervention capable of improving patient outcomes independently. The effectiveness of digital PROM integration may ultimately depend on clinically meaningful interpretation, integration into existing care pathways, and the ability of healthcare teams to translate patient-reported information into appropriate clinical actions.

### 4.5. Implications for Value-Based Healthcare

The growing emphasis on value-based healthcare has strengthened interest in outcome measures that reflect what matters most to patients rather than relying exclusively on traditional clinical endpoints [45,50,61,62]. Within this framework, PROMs offer an opportunity to complement conventional measures of treatment success by capturing symptom burden, functional status, health-related quality of life, and patient-perceived treatment benefit [5,48,57].

However, the present review indicates that the integration of PROMs into value-based AF care remains at an early stage of development. While the conceptual rationale is well established and increasingly supported by international policy initiatives and professional society recommendations [45,50,61,62], empirical evidence demonstrating that PROM-guided care directly improves healthcare value, resource allocation, cost-effectiveness, or reimbursement outcomes remains limited. Most available publications describe implementation frameworks, quality improvement initiatives, or conceptual healthcare models rather than prospective comparative studies specifically conducted in AF populations.

These observations should not be interpreted as evidence against PROM implementation. Rather, they highlight an important transition from instrument validation toward implementation science. Future research should therefore move beyond evaluating psychometric performance alone and focus on whether routine PROM-guided care improves patient-centred outcomes, supports more efficient allocation of healthcare resources, facilitates shared decision-making, and contributes to sustainable value creation within contemporary AF management.

Taken together, the available evidence supports the continued integration of PROMs into AF care pathways while emphasizing that several proposed healthcare system applications—including healthcare quality assessment, value-based care evaluation, and organizational performance measurement—should currently be regarded as promising implementation strategies requiring further prospective validation rather than established standards of routine clinical practice.

Table 5 shows the proposed research agenda for the future development of patient-reported outcome measure (PROM)-guided atrial fibrillation (AF) care. The identified priorities reflect current evidence gaps discussed throughout this review and highlight areas where future clinical, implementation, digital health, and health economic research may facilitate broader integration of PROMs into patient-centered healthcare systems.

Although PROMs are increasingly recognized as key components of value-based healthcare frameworks, empirical evidence supporting their direct impact on value generation in AF remains limited. Most available publications describe conceptual models, policy initiatives, or implementation frameworks rather than prospective studies demonstrating improvements in cost-effectiveness, resource utilization, or patient outcomes attributable to PROM-guided care. Consequently, this application should currently be regarded as an important future direction rather than an established component of routine AF management.

### 4.6. Limitations of the Present Review

Several limitations of the present review should be acknowledged. The study was designed as a narrative implementation review rather than a formal systematic review or meta-analysis. Consequently, the findings reflect thematic interpretation of the available literature rather than quantitative pooled analysis.

Considerable heterogeneity also exists across studies evaluating PROM implementation in AF care, including differences in patient populations, PROM instruments, healthcare settings, digital infrastructure, and implementation approaches. This variability limits direct comparison between studies and complicates interpretation across contexts.

In addition, some implementation concepts discussed in this review particularly those related to digital health integration, AI-assisted interpretation, and healthcare system adaptation have not yet been extensively validated in routine clinical practice. The rapid pace of technological development may further affect the long-term applicability of current implementation models.

Healthcare system variability represents a further contextual limitation. The feasibility and scalability of PROM implementation are likely to differ substantially across institutions, countries, and organisational environments. Taken together, the available evidence supports the continued integration of PROMs into AF care pathways while also highlighting the need for robust prospective implementation studies capable of determining whether these conceptual advantages translate into measurable improvements in clinical outcomes, healthcare quality, and system performance.

## 5. Conclusions and Future Perspectives

### 5.1. Conclusions

Patient-reported outcome measures have evolved from research instruments for measuring health-related quality of life into increasingly important components of patient-centred atrial fibrillation care. The evidence synthesized in this review demonstrates that PROMs complement conventional clinical assessment by capturing symptom burden, functional limitations, emotional well-being, treatment satisfaction, and other dimensions of health that are not adequately reflected by rhythm status or traditional clinical endpoints alone.

Current evidence supports the routine use of validated PROMs for evaluating symptom burden and health-related quality of life, particularly within rhythm-control strategies and longitudinal patient follow-up. At the same time, broader healthcare system applications—including outpatient prioritization, healthcare quality assessment, digital monitoring, and value-based healthcare—remain supported predominantly by observational studies, implementation initiatives, and conceptual frameworks rather than prospective implementation trials. Consequently, these applications should currently be regarded as promising implementation strategies rather than established standards of routine AF care.

Successful PROM implementation may ultimately depend not only on the availability of validated instruments but also on thoughtful integration into clinical workflows, digital infrastructure, multidisciplinary care pathways, and healthcare policy. The 2024 ESC AF-CARE framework provides an important opportunity to incorporate patient-reported outcomes into comprehensive AF management while maintaining an appropriate balance between evidence-based practice and implementation innovation.

### 5.2. Future Research Perspectives

The next phase of PROM research in atrial fibrillation should move beyond psychometric validation toward implementation science. While substantial progress has been achieved in developing reliable and disease-specific PROM instruments, considerably less evidence is available regarding their optimal integration into routine healthcare systems, their influence on clinical decision-making, and their impact on patient outcomes and healthcare performance.

Future studies should prioritize pragmatic prospective implementation trials evaluating whether routine PROM-guided care improves symptom control, patient engagement, healthcare utilization, resource allocation, and long-term clinical outcomes. Particular attention should be given to identifying clinically meaningful PROM thresholds, defining standardized response pathways, and determining how PROM data can most effectively support shared decision-making without increasing clinician workload or administrative burden.

Digital health technologies are expected to play an increasingly important role in this process. Integration of electronic PROMs with wearable devices, remote rhythm monitoring, electronic health records, and artificial intelligence–supported clinical decision systems may enable more continuous and individualized assessment of patient health status. However, future investigations should evaluate not only technological feasibility but also clinical effectiveness, cost-effectiveness, interoperability, data governance, and equitable access across diverse healthcare environments.

Further refinement of AF-specific PROMs is also warranted. Existing instruments primarily focus on symptom burden and health-related quality of life, whereas domains such as cognitive function, sleep quality, fatigue, caregiver burden, treatment burden, work productivity, and social participation remain incompletely represented. Development and validation of next-generation PROM instruments capable of capturing these broader dimensions may improve the comprehensiveness of patient-centred assessment.

Finally, future research should evaluate the contribution of PROMs to value-based healthcare and integrated AF management. As healthcare systems increasingly emphasize outcomes that matter to patients, prospective studies should determine whether systematic PROM implementation can support healthcare quality improvement, benchmarking, multidisciplinary care coordination, and more efficient allocation of healthcare resources within the AF-CARE framework. Such evidence may be essential to establish PROMs not only as patient-reported outcome measures but also as operational instruments capable of supporting sustainable, patient-centred, and value-based atrial fibrillation care.

## Figures and Tables

**Table 1:** Characteristics and implementation considerations of commonly used patient-reported outcome measures (PROMs) in atrial fibrillation care.

Instrument	Type	Main Domains Assessed	Major Strengths	Important Limitations	Respondent Burden	Approximate Administration Time	Digital Adaptability	Potential Clinical and Healthcare System Utility
AFEQT (Atrial Fibrillation Effect on Quality-of-Life Questionnaire)	AF-specific	Symptoms, daily activities, treatment concern, treatment satisfaction	Widely validated; highly sensitive to AF-related quality-of-life changes; extensively used in rhythm-control and ablation studies	Limited assessment of cognition, sleep disturbance, productivity, and broader psychosocial burden	Low–Moderate	≈5–7 min	Excellent (fully compatible with electronic PROM platforms)	Longitudinal monitoring; treatment response assessment; outpatient follow-up; symptom-guided reassessment; digital AF care pathways
AFSS (University of Toronto Atrial Fibrillation Severity Scale)	AF-specific	Symptom severity, episode frequency, global AF burden	Practical symptom-oriented assessment; suitable for repeated rhythm-control follow-up	Less comprehensive multidimensional quality-of-life assessment	Moderate	≈5–10 min	Good	Symptom tracking; rhythm-monitoring programmes; serial outpatient assessment
EQ-5D	Generic	Mobility, self-care, usual activities, pain/discomfort, anxiety/depression	Broad comparability across diseases; widely used in health economic analyses; supports QALY estimation	Limited sensitivity to AF-specific symptom changes	Very low	≈2–3 min	Excellent (widely available electronically)	Value-based healthcare; health economic evaluation; registry studies; population health assessment
SF-36	Generic	Physical functioning, vitality, emotional well-being, social functioning, general health	Comprehensive multidomain assessment; extensive international validation	Longer questionnaire; reduced responsiveness to short-term AF symptom changes	High	≈10–15 min	Good	Comprehensive quality-of-life assessment; longitudinal observational studies; research applications
WPAI (Work Productivity and Activity Impairment Questionnaire)	Generic/Work-related	Work productivity, absenteeism, presenteeism, activity impairment	Captures occupational burden and indirect socioeconomic impact	Not disease-specific; limited assessment of clinical AF symptoms	Low	≈3–5 min	Excellent	Assessment of productivity loss; indirect healthcare costs; socioeconomic impact analyses

**Table 2:** Evidence-supported and emerging operational applications of PROMs in atrial fibrillation care.

Operational Role	Description	Potential Clinical and Healthcare System Impact	Evidence Status
Symptom assessment	Systematic evaluation of symptom severity, frequency, and variability using validated PROM instruments	Supports individualized symptom assessment, treatment planning, and longitudinal follow-up	Evidence-supported
Health-related quality of life (HRQoL) monitoring	Serial assessment of patient-reported quality of life during follow-up	Facilitates evaluation of treatment response and patient-centred outcomes	Evidence-supported
Shared decision-making	Incorporation of patient-reported outcomes into discussions regarding treatment options	Enhances patient engagement, communication, and individualized therapeutic decision-making	Evidence-supported
Monitoring treatment response	Repeated PROM assessment after rhythm-control interventions, catheter ablation, or pharmacological therapy	May improve recognition of symptom changes and perceived treatment effectiveness	Emerging evidence
Longitudinal outpatient monitoring	Continuous collection of PROM data during routine outpatient care	May facilitate earlier identification of symptom deterioration and guide follow-up intensity; prospective AF-specific evidence remains limited	Emerging evidence
Outpatient prioritization	Use of PROM trajectories to identify patients requiring earlier clinical reassessment	Potentially supports prioritization of clinical resources and individualized follow-up scheduling	Future implementation opportunity
Healthcare quality assessment	Aggregated PROM data used to evaluate healthcare performance and patient-centred quality indicators	May complement traditional quality metrics and support continuous quality improvement	Future implementation opportunity
Healthcare resource allocation	Population-level PROM analysis to identify unmet needs and optimize healthcare planning	Potential application for resource prioritization and service development; requires prospective validation	Future implementation opportunity
Value-based healthcare (VBHC)	Integration of PROMs into value-based healthcare frameworks focusing on outcomes meaningful to patients	May support outcome measurement and value assessment within AF care pathways	Future implementation opportunity
Digital health integration and AI-assisted decision support	Integration of electronic PROMs with digital health platforms, wearable technologies, and future AI-assisted clinical decision support	Promising future strategy for personalized AF management; currently supported mainly by conceptual and early implementation literature	Future implementation opportunity

**Table 3:** Integration of patient-reported outcome measures (PROMs) across the 2024 ESC AF-CARE pathway.

AF-CARE Component	Potential Role of PROMs	Representative Evidence	Current Level of Evidence	Remaining Evidence Gaps
C—Comorbidity and cardiovascular risk management	Identification of symptom burden related to multimorbidity; assessment of treatment priorities; monitoring the patient-perceived impact of concomitant diseases	Observational cohort studies, integrated AF care programmes, ESC Guidelines 2024	Moderate	Limited evidence regarding routine PROM-guided management of multimorbidity and cardiovascular risk in AF
A—Avoid stroke and thromboembolism	Assessment of treatment satisfaction, anticoagulation-related quality of life, patient preferences, and shared decision-making	Observational studies, patient preference research, guideline recommendations	Low–Moderate	Few prospective studies evaluating whether PROM-guided anticoagulation management improves adherence or clinical outcomes
R—Reduce symptoms by rate and rhythm control	Quantification of symptom burden, assessment of treatment response, evaluation of health-related quality of life following antiarrhythmic therapy or catheter ablation	AFEQT validation studies; CABANA; CAPTAF; ESC Guidelines 2024	High	Limited evidence supporting routine PROM-guided adjustment of rhythm-control strategies
E—Evaluation and dynamic reassessment	Longitudinal symptom monitoring; identification of functional deterioration; support for follow-up prioritization; integration with digital monitoring platforms	Observational studies, digital health pilot programmes, integrated care initiatives	Moderate	Prospective AF-specific implementation studies evaluating clinical effectiveness and healthcare impact remain lacking
Patient engagement (cross-cutting principle)	Shared decision-making; individualized treatment goals; communication between patients and multidisciplinary teams; evaluation of treatment success from the patient perspective	ESC 2024 AF Guidelines; integrated care programmes; implementation studies	Moderate	Standardized implementation strategies and comparative effectiveness studies remain limited

**Table 4:** Major Barriers to PROM Implementation in Atrial Fibrillation Care and Potential Mitigation Strategies.

Implementation Barrier	Potential Clinical and Organizational Impact	Possible Mitigation Strategies
Questionnaire burden and patient fatigue	Reduced long-term adherence and incomplete reporting	Use of shorter adaptive PROM formats; selective longitudinal assessment
Clinician workload and time constraints	Reduced integration into routine consultations	Automated scoring systems; workflow-aligned digital platforms
Limited interpretability of PROM changes	Difficulty translating scores into clinical decisions	Development of clinically actionable interpretation frameworks
Poor electronic health record interoperability	Fragmented documentation and inefficient data utilization	Standardized digital integration pathways and interoperable systems
Digital inequality and limited patient digital literacy	Unequal access to remote monitoring and electronic PROM collection	Hybrid paper–digital approaches; patient education support
Lack of organizational support	Inconsistent implementation and low sustainability	Institutional implementation strategies and leadership engagement
Variability between healthcare systems	Uneven implementation quality and limited comparability	Flexible implementation models adapted to local infrastructure
Overreliance on numerical scoring	Risk of reducing individualized patient assessment	Integration of PROMs into broader clinical evaluation rather than isolated score interpretation
Limited multidisciplinary coordination	Underrecognition of psychosocial and functional burden	Inclusion of nursing, psychology, rehabilitation, and primary care support
Data privacy and ethical concerns	Reduced trust in digital PROM platforms	Transparent governance frameworks and secure data management systems

**Table 5:** Research Agenda for Future Development of PROM-Guided Atrial Fibrillation Care.

Research Domain	Current Knowledge Gap	Proposed Research Priority	Potential Impact on AF Care
PROM content development	Current instruments incompletely capture cognitive symptoms, sleep quality, sexual health, and caregiver burden	Develop multidomain AF-specific PROM modules	More comprehensive assessment of patient burden
Longitudinal implementation	Limited evidence regarding optimal assessment frequency and timing	Prospective longitudinal implementation studies	Improved monitoring and personalized follow-up
Digital health integration	Variable interoperability between PROM platforms and electronic health records	Develop interoperable digital PROM ecosystems	More efficient clinical workflow and data integration
Artificial intelligence	Limited validation of AI-assisted PROM interpretation	Evaluate explainable AI models for clinical decision support	Earlier identification of deterioration and individualized care
Health economics	Limited evidence linking PROM implementation with healthcare resource utilization and cost-effectiveness	Conduct prospective cost-effectiveness and budget impact analyses	Stronger evidence for value-based healthcare adoption
Implementation science	Few pragmatic implementation studies in routine clinical practice	Evaluate workflow integration, clinician acceptance, and organizational sustainability	Improved scalability of PROM-guided care
Smaller healthcare systems	Limited evidence from low-resource and underrepresented healthcare environments	Regional implementation and validation studies	Greater international applicability and equity
Multidisciplinary care	Unclear integration of PROMs across cardiology, primary care, nursing, psychology, and rehabilitation	Develop multidisciplinary implementation pathways	Better continuity of patient-centered AF care

## Data Availability

No new data were created or analyzed in this study. Data sharing is not applicable to this article.

## Supplementary Materials

The following supporting information can be downloaded at https://www.mdpi.com/article/10.3390/healthcare14131904/s1. Supplementary Table S1: Database-specific literature search strategies used for the narrative implementation review.

## Abbreviations

The following abbreviations are used in this manuscript:

AF	Atrial fibrillation
AF-CARE	Atrial fibrillation-CARE integrated management pathway
AFEQT	Atrial Fibrillation Effect on Quality-of-Life Questionnaire
AFSS	Atrial Fibrillation Severity Scale
AI	Artificial intelligence
CFIR	Consolidated Framework for Implementation Research
EHR	Electronic health record
EHRA	European Heart Rhythm Association
ESC	European Society of Cardiology
EQ-5D	EuroQol five-dimensional questionnaire
HRQoL	Health-related quality of life
NPT	Normalization Process Theory
PREMs	Patient-reported Experience Measures
PROMs	Patient-reported outcome measures
QALY	Quality-adjusted life year
RE-AIM	Reach, Effectiveness, Adoption, Implementation, Maintenance
SF-36	Short Form Health Survey
VBHC	Value-based healthcare
WPAI	Work Productivity and Activity Impairment Questionnaire
