# Supplementary material for: Integrating Patient-Reported Outcomes into Atrial Fibrillation Care Pathways: Implementation Challenges, Health System Implications, and Future Directions
Source: Healthcare (Basel). 2026 Jun 30;14(13):1904. doi: 10.3390/healthcare14131904 (PMC13361731; doi:10.3390/healthcare14131904)
Supplement: Supplementary file 1 [file healthcare-14-01904-s001.zip › healthcare-4364257-supplementary.pdf]

Supplementary Table S1

Database-Specific Search Strategies

Table S1. Database-specific literature search strategies used for the narrative implementation review.

Database	Search strategy	Filters	Records identified
**PubMed/MEDLINE**	(("atrial fibrillation"[Title/Abstract] OR AF[Title/Abstract]) AND ("patient-reported outcome*"[Title/Abstract] OR PROM*[Title/Abstract] OR AFEQT[Title/Abstract] OR AFSS[Title/Abstract] OR "quality of life"[Title/Abstract]) AND (implementation[Title/Abstract] OR "healthcare system*"[Title/Abstract] OR "patient-centered care"[Title/Abstract] OR "value-based healthcare"[Title/Abstract] OR "digital health"[Title/Abstract] OR "remote monitoring"[Title/Abstract] OR "health policy"[Title/Abstract]))	English; 1 January 2000–31 March 2025	**320**
**Scopus**	TITLE-ABS-KEY(("atrial fibrillation" OR AF) AND ("patient-reported outcome*" OR PROM OR PROMs OR AFEQT OR AFSS OR "quality of life") AND (implementation OR "healthcare system*" OR "patient-centered care" OR "value-based healthcare" OR "digital health" OR "remote monitoring" OR "health policy"))	English; Articles; Reviews; 2000–2025	**421**
**Google Scholar**	Supplementary searches using combinations of *atrial fibrillation*, *patient-reported outcomes*, *quality of life*, *implementation*, *digital health*, *value-based healthcare*, and related terms	Supplementary searches only	Not used for quantitative record identification

Supplementary Table S1. Database-specific search strategies used to identify publications included in the narrative implementation review. Google Scholar was used only as a supplementary source for identifying recently published literature, guideline-related documents, citation tracking, and potentially relevant grey literature.
